# Strategies to Combat Heat Stress in Broiler Chickens: Unveiling the Roles of Selenium, Vitamin E and Vitamin C

**DOI:** 10.3390/vetsci7020071

**Published:** 2020-06-01

**Authors:** Majid Shakeri, Ehsan Oskoueian, Hieu Huu Le, Mehdi Shakeri

**Affiliations:** 1 Department of Medicine, The University of Washington, Seattle, WA 98195, USA; mshakeri@uw.edu; 2 Mashhad Branch, Agricultural Biotechnology Research Institute of Iran, Agricultural Research, Education, and Extension Organization, Mashhad 511, Iran; 3 Faculty of Animal Sciences, Vietnam National University of Agriculture, Trau Quy, Gia Lam, Hanoi 131004, Vietnam; lhhieu.hua@gmail.com; 4 Department of Animal Science and Agriculture, Ferdowsi University of Mashhad, Mashhad 511, Iran; mehdi.shakeri95@gmail.com

**Keywords:** functional feed additive, oxidative stress, vitamins, free radicals

## Abstract

Heat stress compromises efficient poultry production by impairing growth performance and increasing mortality. Mechanisms to dissipate excess heat divert energy from efficient production. This includes increased energy expenditure for respiration, oxidative stress and micronutrient absorption. The fortification of diets with particular feed additives has been known as one of the most important approaches to minimize the negative impacts of heat stress on broiler production. In this context, the promising functional feed additives appeared to be selenium and vitamins E and C. The fortification of broiler diets with these feed additives has been proven to enhance the function of vital organs, immune system response and growth performance of broilers under heat stress. The current review highlights recent successful experiences in the alleviation of heat stress symptoms in broilers using the above-mentioned additives. Selenium and vitamins E and C enhanced production performance in broiler chickens challenged with acute heat stress. The combination of these additives, by employing multiple mechanisms and through synergistic effects, improves heat stress symptoms more efficiently than their individual forms. Emerging literature reveals that selenium and vitamins E and C are involved in close interactions to protect proteins and lipids from oxidative damage and boost immune system function.

## 1. Introduction

**Figure 1:**
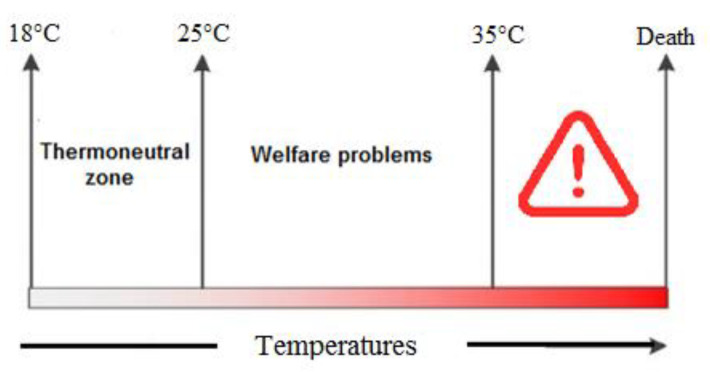
The temperature between the lower critical and upper critical temperatures (thermoneutral zone, 18–25 °C) is defined as the temperature zone in which chickens are able to keep their body temperature constant with the help of physical heat regulation (normal behaviors). In the critical zone, chickens are unable to keep their body temperature constant, resulting in welfare problems such as fast panting, physical tiredness (26–35 °C) and death (<36 °C) [7].

A high environmental temperature is one of the most important factors that causes heat stress among chickens and negatively affects poultry production [1]. Usually, the optimum temperature for growing broilers is 18 to 22 °C [2], and any temperature higher than this range could cause heat stress [3]. Heat stress normally happens in the summer season when there is a negative balance between the environmental temperature and body heat production. In fact, chickens are confronted with three different ranges of temperature zones—the comfort zone, critical zone and upper critical zone. In the comfort zone (18–25 °C), chickens can maintain their body temperature with minimum effort, while in the critical zone (26–35 °C), maintaining body temperature requires the help of physical heat regulation. Furthermore, in the upper critical zone (higher than 35 °C), chickens cannot dissipate their body’s heat and physiological disorders appear following multi-organ dysfunction, resulting in death [4] (Figure 1).

Generally, under heat stress condition, chickens attempt to maintain their body temperature within the comfort zone to ensure the function of all vital organs. Thereby, they limit feed intake, walking and standing while increasing resting time, drinking and panting [5]. Under chronic heat stress, the continuous panting of the bird could alter the blood pH leading to respiratory alkalosis. Moreover, the changes in the blood pH impair the immune system’s function and the body’s hormonal activity [6].

It is known that blood flow plays an important role in controlling the body’s temperature. Under conditions of heat stress, the blood flow distribution is diverted from visceral to peripheral capillary beds for a rapid decrease in the body’s temperature. On the other hand, the reduced visceral blood flow may cause hypoxia in the gastrointestinal tissues [8]. Hypoxia occurs when the body is deprived of an adequate oxygen supply such as during metabolism. The hypoxia in the gastrointestinal tract, particularly intestinal tissues, resulted in oxidative stress damage and increased permeability to pathogens and their related endotoxins [9,10]. Oxidative stress impairs the intestinal immune system’s function, promotes mucosal and villus degeneration and causes enteric infections [11,12]. As a consequence, the digestion and absorption of the nutrients is interrupted, which could contribute to bone abnormalities, skeletal disorders and lameness [13].

It has been indicated that heat stress could alter the functions of the hypothalamic-pituitary axis and orthosympathic nervous system. As a result, the concentrations of thyroid hormones such as triiodothyronine (T_3_) and thyroxine (T_4_) were elevated, whereas the serum corticosterone concentration decreased. Emerging recent literature revealed that heat stress decreased feed intake and increased feed conversion ratios and mortality [14]. All these negative impacts resulted in low broiler production efficiency with high, significant economic losses [15]. Trace elements such as selenium are very important in the high-stress production condition. Recent findings indicated that both the level and the source of trace elements could play an important role in optimizing the production level, product quality and health status of the birds and economic returns [16].

A survey of recent progress in heat stress management in broilers revealed the potential applications of bioactive compounds (selenium and vitamins C and E) as a feasible approach to combat heat stress symptoms. Therefore, due to the importance of these functional bioactive compounds in heat stress management, this review aimed to highlight the detailed information on how these bioactive compounds may contribute to alleviate heat stress symptoms in the broiler industry.

## 2. Bioactive Compounds in Heat Stress

### 2.1. Ascorbic Acid

Ascorbic acid, ascorbate (the anion of ascorbic acid) or vitamin C is a water-soluble antioxidant compound, which protects cells against oxidative damage and improves immune system function [17,18]. In fact, vitamin C is not a part of any metabolic pathway, but it is an essential co-factor in many enzymatic reactions such as the synthesis of collagen, carnitine and catecholamine, and the metabolism of microsomes or the synthesis and catabolism of tyrosine.

Vitamin C activates the lysyl hydroxylase, prolyl-4-hydroxylase and prolyl-3-hydroxylase enzymes, which are involved in the conversion of peptide-bound proline and lysine into hydroxyproline and hydroxylysine. These conversions are essential for the formation of collagen. The collagen is essential for the development and maintenance of blood vessels, scar tissue, cartilage and bones. The fibril networks of collagen are also critical in bone formation through mineralization with hydroxyapatite under the influence of active metabolites of vitamin D_3_. It has also been reported to improve bone-breaking strength through increased collagen synthesis, the enhanced binding capacity of calcium-binding proteins and the mineralization process [18,19,20].

In the carnitine pathway, the protein is degraded to 3-hydroxy-6-N-trimethyllysine by 6-*N*-trimethyllysine hydroxylase, and the resulting product, into gamma-butyrobetaine hydroxylase and glycine. Then, gamma-butyrobetaine hydroxylase is hydroxylated to carnitine. In this process, the conversion of trimethyllysine to 3-hydroxy-trimethyllysine and 4-butyrobetaine to carnitine occurs in the presence of vitamin C. Carnitine plays a major role in essential metabolic pathways, imports activated fatty acids into the mitochondrion for ATP generation and functions as an osmolyte in the cytoplasm [21].

Vitamin C is a co-factor of dopamine beta hydroxylase, which participates in the conversion of dopamine to norepinephrine in neural tissues [22]. Norepinephrine levels generally increase during stress and increase heart rate and blood pressure, increase blood glucose, increase blood flow to skeletal muscle and reduce blood flow to and the motility of the gastrointestinal tract.

In addition to the biosynthesis of norepinephrine, vitamin C appeared to be essential in the bioconversion of tyrosine to other catecholamines such as dopamine, noradrenaline and adrenaline [23,24,25]. In fact, tyrosine is a precursor to neurotransmitters and increases plasma neurotransmitter levels under stressful conditions. Various studies have indicated that supplementation with tyrosine and vitamin C during stress could reduce stress hormones and weight loss in animal trials [26,27]. The available information has also confirmed that vitamin C is not only involved in hormone biosynthesis but also increases their stability and activity through an amidation process with the help of an enzyme called peptidylglycine alpha-amidating monooxygenase. Vitamin C has also been shown to regulate the body’s temperature, synthesis of 1,25-dihydroxy vitamin D, and function of the immune system. Vitamin C is present in high concentrations in the immune cells, and it is depleted quickly during stress conditions. It is not exactly clear how vitamin C boosts immune system function, but some of available evidence indicates the effects of vitamin C on phagocytes, the production of cytokines, lymphocytes and the number of cell adhesion molecules in monocytes [28,29].

Vitamin C may act as co-antioxidant with other antioxidants by synergistic effects. In the research conducted by Doba et al. [30], the antioxidant activity of vitamin E increased in the presence of vitamin C through reducing tocopheroxy radicals back to their active form of vitamin E. Emerging literature revealed that adult poultry are capable of synthesizing vitamin C to meet their requirements in normal conditions. However, it has been found that their requirements increase during stress, and several studies have reported the beneficial effects of the dietary supplementation of poultry feed with ascorbic acid [17,31]. Dietary supplementation with vitamin C limited and alleviated stress metabolic signs, improved performance, enhanced immunological status and reduced mortality. The literature survey indicated that the optimum response in terms of the growth performance, feed conversion ratio, feed efficiency, survival rate and carcass quality in broilers under heat stress appeared to occur with average supplements of 250 mg/kg vitamin C. However, the required vitamin C in laying hens to observe optimum feed efficiency, egg production and egg quality under heat stress is about 200–500 mg/kg [17,32,33,34,35,36].

There is a close relationship between vitamin C with vitamin E, whereas both have positive impacts on the immune system by enhancing antibody production, macrophage activity and humoral immunity in broiler chicks. Therefore, a combination of these two vitamins could improve the immune response and performance of broiler chickens.

### 2.2. Vitamin E

Vitamin E is a group of compounds containing both tocopherols and tocotrienols, which are fat-soluble with antioxidant properties [37]. Vitamin E protects the cell membranes and tissues from lipoperoxidative damage induced by free radicals [38,39] and acts as a cellular enzymatic activity regulator [40]. Alpha-tocopherol is the most biologically active form of vitamin E, which is involved in the glutathione peroxidase pathway and protects organisms from oxidative damage by reacting with lipid radicals produced in the lipid peroxidation reaction. The oxidized alpha-tocopherol radicals recycle back to the active reduced form through reduction by other antioxidants such as ascorbate. Ascorbic acid acts as an antioxidant or a prooxidant and helps to maintain vitamin E levels through reducing the degradative metabolism of vitamin E and then increases the antioxidant effectiveness.

The fat-soluble nature of vitamin E renders it as a suitable cell membrane antioxidant against oxidative damage. Moreover, vitamin E modulates the activity of protein kinase C (PC) and controls smooth muscle growth and proliferation. Vitamin E activates a dephosphorylation enzyme called protein phosphatase 2, which cuts phosphate groups from PC and results in the inactivation of PC and smooth muscle growth inhibition [41,42]. Poultry vitamin E requirements should be provided for through dietary supplementation as they are not capable of vitamin E synthesis. Under stress conditions, particularly heat stress, the levels of hormones like corticosterone and catecholamines increase and lipid peroxidation in cells membranes is initiated.

Vitamin E has also been reported to protect lymphocytes, macrophages and plasma cells against oxidative damage and improves their survival, proliferation and function. Thus, dietary supplementation with vitamin E under stress conditions enhances the immune response [43]. Emerging literature reveals that the addition of vitamin E at an average concentration of 250 mg/kg is a feasible protective practice to alleviate the symptoms of heat stress and may result in optimum performance and improved meat quality in broiler chickens [44,45]. However, this concentration for layers is 125–250 mg/kg, which leads to an enhanced immune response, egg production and feed conversion ratio [43,44,46]. The improved egg production under heat stress conditions appeared to be through protecting the liver from lipid peroxidation and cell membrane damage, which resulted in plasma egg yolk precursors increasing, including very low density lipoprotein and vitellogenin [47]. It could facilitate the regeneration of oxidative stress-damaged tissues [48] and enhances neurological function [49]. Heat stress increases the concentration of malonyldialdehyde in the serum and liver, whereas vitamin E reduces the synthesis of malonyldialdehyde in the liver by acting against lipid peroxidation and cell damage [50] and results in the improvement of the chicken’s performance [51,52].

Phenolics, flavonoids, saponins and alkaloids are major plant bioactive compounds with potential antioxidant activity. Earlier research indicated that plant bioactive compounds with antioxidant activity work synergistically with vitamin E and intensify the antioxidant potential of vitamin E [53]. Besides phytogenics, zinc is an element that is important in carbohydrate metabolism, protein synthesis [54,55] and adjusting insulin levels, which are important in glucose pathway [56,57]; the production of epithelial cells, which are one of the intestinal barriers against harmful external organisms [55]; and T-cell and immune system functions [58,59]. Under heat stress conditions, zinc (30–60 mg/kg) has synergistic effects with vitamin E, and their combination positively affects laying hens’ health and egg production [60,61,62] and the performance of broilers [63,64]. The combination of vitamin E and zinc is considered as an important antioxidant shield for broilers against heat stress [65,66].

Furthermore, there is also a close relationship between selenium and vitamin E; both act to improve the immune system. It needs to be mentioned that different sources of selenium provide different alterations in the immune system.

### 2.3. Selenium

Selenium is an essential micronutrient and can be found in two forms as inorganic (selenate and selenite) and organic forms (selenomethionine and selenocysteine). Selenium functions as a co-factor for antioxidant enzymes including glutathione peroxidase, superoxide dismutase and thioredoxin reductase. Glutathione peroxidase removes reactive oxygen species, protects cells against oxidative stress damage [67], and prevents lipid and protein oxidation [68]. Furthermore, selenium is considered as a co-factor for iodothyronine deiodinase enzymes, which are involved in the activation or inactivation of the initially released hormone T_4_ to T_3_ or reverse triiodothyronine (rT_3_). Thus, selenium, by the indirect regulation of T_3_ and T_4_ production, affects the basal metabolic rate, protein synthesis, and the metabolism of fat, carbohydrate, protein and vitamins. It is known that under physiological, pathological or environmental stimuli, the synthesis of thyroid hormones is impaired and supplementing with selenium effectively regulates the synthesis of thyroid hormones and restores body homeostasis.

Initially, the organic or inorganic forms of selenium compound are transformed to hydrogen selenide. Selenomethionine could act as methionine functions or be transformed to selenocysteine via the trans-sulfuration pathway. Then, selenocysteine may be transformed to hydrogen selenide. Selenate is reduced to selenite by glutathione and then hydrogen selenite. Selenophosphate synthetase converts the hydrogen selenite to selenophosphate. Then, it reacts with L-seryl-tRNASec to form l-selenocysteinyl-tRNASec, which then further forms the selenoproteins. A selenoprotein is any protein containing selenocysteine, and common selenoproteins are glutathione peroxidase and thioredoxin reductase, which are involved in oxidative damage and thyroid hormone metabolism, respectively. The function of the immune system has also been enhanced in the presence of these two selenoproteins [69,70].

Early studies in broilers indicated that dietary supplementation with selenium at doses ranging from 0.4 to 1 mg/kg significantly enhanced growth performance when chickens were exposed to heat stress [71,72]. These results were further attributed to the effects of selenium on thyroid hormone metabolism, DNA synthesis, cellular antioxidant levels and immune system responses [73,74,75].

## 3. Conclusions

Considering new techniques to cope with heat stress, which has an adverse influence on the performance and meat quality during the rearing period, is a main aim of the poultry industry. In order to enhance chickens’ tolerance to high temperature, understanding the functions of different supplementations and manipulating diets seem to be promising methods to reduce the adverse effects of heat stress quickly. The synergistic effects of selenium and vitamins E and C enhance growth performance in broiler chickens challenged with acute heat stress, employing multiple mechanisms. Emerging literature reveals that selenium and vitamins E and C have close interactions to protect proteins and lipids from oxidative damage, and a combination of them can be considered as an important solution to cope with heat stress. Both vitamins C and E play an important role in tissues: scavenging oxygen radicals, resulting in the improvement of the immune system. Furthermore, vitamin C regenerates vitamin E by reducing the vitamin E radicals formed. Meanwhile, selenium is involved in the glutathione pathway, which is part of the ascorbate cycle.
